# Improved Surface Display of Human Hyal1 and Identification of Testosterone Propionate and Chicoric Acid as New Inhibitors

**DOI:** 10.3390/ph13040054

**Published:** 2020-03-26

**Authors:** Isabelle Lengers, Fabian Herrmann, Marc Le Borgne, Joachim Jose

**Affiliations:** 1 Institute of Pharmaceutical and Medicinal Chemistry, PharmaCampus, Westfälische Wilhelms-Universtität Münster, 48149 Münster, Germany; isabelle.lengers@uni-muenster.de; 2 Institute of Pharmaceutical Biology and Phytochemistry, PharmaCampus, Westfälische Wilhelms-Universtität Münster, 48149 Münster, Germany; shermanic@uni-muenster.de; 3 Université de Lyon, Université Claude Bernard Lyon 1, Faculté de Pharmacie—ISPB, EA 4446 Bioactive Molecules and Medicinal Chemistry, SFR Santé Lyon-Est CNRS UMS3453—INSERM US7, 8 Avenue Rockefeller, F-69373 Lyon CEDEX 8, France; marc.le-borgne@univ-lyon1.fr

**Keywords:** hyaluronic acid, hyaluronidase, hyal1, autodisplay, inhibitors

## Abstract

Degradation of high molecular weight hyaluronic acid (HA) in humans is mainly catalyzed by hyaluronidase Hyal1. This enzyme is involved in many pathophysiological processes and therefore appears an interesting target for drug discovery. Until now, only a few inhibitors of human Hyal1 are known due to obstacles in obtaining active enzymes for inhibitor screening. The aim of the present work was to provide a convenient enzyme activity assay and show its feasibility by the identification of new inhibitors. By autodisplay, *Escherichia coli* F470 can present active Hyal1 on its surface. In this study, the inducible expression of Hyal1 on the cell surface of *E. coli* under the control of a rhamnose-dependent promoter (P_rha_) was performed and optimized. Enzyme activity per single cell was increased by a factor of 100 compared to the constitutive Hyal1 surface display, as described before. An activity of 6.8 × 10^−4^ mU per single cell was obtained under optimal reaction conditions. By this modified activity assay, two new inhibitors of human Hyal1 were identified. Chicoric acid, a natural compound belonging to the phenylpropanoids, showed an IC_50_ value of 171 µM. The steroid derivative testosterone propionate showed and IC_50_ value of 124 ± 1.1 µM. Both values were in the same order of magnitude as the IC_50_ value of glycyrrhizic acid (177 µM), one of the best known inhibitors of human Hyal1 known so far. In conclusion, we established a new enzyme activity assay for human Hyal1 and identified new inhibitors with this new assay method.

## 1. Introduction

Hyaluronic acid (HA) is the major glycosaminoglucan of the extracellular matrix (ECM) [1]. This polysaccharide can exhibit molecular weights up to 10^5^–10^7^ kDa. It consists of repeating disaccharide units [(1,3)-β-D-GlcNAc-(1,4)-β-D-GlcA]. The interaction of high molecular weight (HMW) HA (>20 kDa) and water molecules lead to high viscosity and elasticity [2,3]. HA’s physiological and pathophysiological functions depend on its level of fragmentation. HMW HA plays an important role in water and plasma protein homeostasis due to its physicochemical properties. It serves as a barrier in the connective tissue, skin and joints that filter molecules and is known to have space-filling effects [3]. Depending on its molecular weight, HA is also relevant in different phases of the wound healing process [4]. The metabolism of HA is catalyzed by specific hyaluronidases and HA synthases. Low molecular weight (LMW) HA fragments resulting from hyaluronidases were shown to have inflammatory, angiogenic and immunosuppressive properties [5,6]. Such pathophysiological effects have not only been observed in cancer, but also in chronic inflammatory diseases. Degradation of HA is catalyzed by specific hyaluronidases (endo-β-N acetyl-D-hexosaminidases). The human genome encodes for six hyaluronidases located on two chromosomes, including Hyaluronidase 1 (Hyal1), Hyaluronidase 2 (Hyal2), Hyaluronidase 3 (Hyal3), Hyaluronidase 4 (Hyal4), Sperm adhesion molecule 1 (SPAM1/PH20) and a pseudogene *HYALP1* encoding for an mRNA, which is not translated to a protein [7,8]. While enzymatic activity for Hyal3 was never confirmed, chondroitin sulfate appeared to be the main substrate for Hyal4 [9,10]. PH20, a membrane-bound hyaluronidase, predominantly expressed on sperms. It contributes to the fertilization of the oocyte by degradation of the HA rich cumulus [11]. The main HA degradation in the human body is due to Hyal1 and Hyal2 activity. Where Hyal2 degrades HA fragments beyond 20 kDa, Hyal1 catalyzes the hydrolyzing of 20 kDa HA into LMW HA fragments with pathophysiological effects, as described above. Hyal1 is usually expressed in many different types of cells. It is both a lysosomal and a secreted enzyme, located in the ECM. Its expression level was shown to be elevated in different types of cancer, e.g., prostate, bladder, or breast cancer [12,13,14,15]. The level of expression was found to correlate with tumor stage and grade in bladder cancer and could therefore be used as diagnostic and prognostic biomarker [16]. Inhibition of Hyal1 results in a decrease in angiogenic, inflammatory and immunosuppressive LMW HA fragments. This makes Hyal1 an interesting target for the treatment of cancer, in particular, bladder, prostate or breast cancer and for the treatment of inflammatory diseases like arthritis or gingivitis [17,18,19]. In the past, most studies on the identification of hyaluronidase inhibitors were performed with bovine testicular hyaluronidase (BTH). A number of different inhibitors have been identified. These compounds differ in structure, source and mode of inhibition. Natural compounds and whole plant extracts, as well as synthetically compounds and polymers have been described. Various flavonoids were screened for BTH inhibition; apigenin was investigated several times with differing results (67% inhibition at 250 µM or 57% inhibition at 1 mM) [20,21]. Further well-known BTH inhibitors are L-ascorbic-acid-6-hexadecanoate (Vcpal) (IC_50_ = 56 µM) and glycyrrhizic acid (IC_50_ 3 - 1300 µM), which are used as reference compounds [22,23,24]. It cannot be ignored, at this point, that observed differences in the degree of inhibition were due to the different enzyme activity assays applied.

Despite Hyal1 being an interesting target, the number of human Hyal1 inhibitors known so far is very limited. This appears to be due to restricted access to the enzyme for testing. Extraction of the enzyme using human urine or plasma is a complex process and leads to low enzyme yields [25,26,27]. Recombinant expression of Hyal1 in *E. coli* led to the formation of inclusion bodies, requiring refolding *in vitro* after purification, finally leading to low enzyme yield and activity (0.11 U/mg). Expression and purification using DS-2 expression system led to a Hyal1 with higher activity (8.6 U/mg), but, for this method, several chromatographic steps for purification were necessary [28]. The protein amount finally obtained was sufficient only for a few tests, including inhibition testing of reference inhibitors such as Vcpal and glycyrrhizinic acid. The IC_50_ values of Vcpal and glycyrrhizic acid were determined to be > 50 µM and 26 or 39.4 µM, respectively [24,28].

Since recombinant expression of Hyal1 is still a bottleneck for the identification of inhibitors, we have developed an assay based on the constitutive expression of Hyal1 on the surface of *E. coli* F470. Production of immobilized Hyal1 on the surface of *E. coli* F470 enables immediate compound screening without further steps of Hyal1 isolation and purification. In former studies, the IC_50_ value of glycyrrhizic acid was determined to be 177 µM [29]. In addition, we were able to identify the aqueous extracts of marshmallow roots (*Althaea officinalis*) (IC_50_ = 7.7 mg/mL) as new Hyal1 inhibitors by application of this autodisplay-based assay [30]. 

In this study, we were able to increase the surface display of Hyal1 on *E. coli* F470 and the activity per single cell by a factor of 100 in replacing the constitutive promotor with a rhamnose-inducible promotor and by optimizing the reaction conditions. *Via* the improved enzyme activity test, we were able to identify two new potent inhibitors with different scaffolds.

## 2. Results

### 2.1. Surface Expression of Hyal1

**Figure 1:**
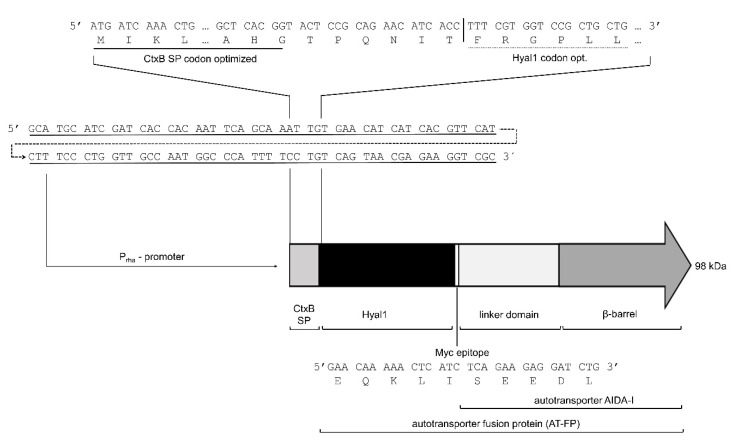
Schematic description of main features of pAIDAI_rha_-Hyal1 expression vector. DNA and amino acid sequences are given for parts of cholera toxin β-subunit signal peptide (CtxB SP), Hyal1 and the myc epitope, as well as the DNA sequence of the P_rha_ promoter used for inducible expression.

In previous studies on the surface display of Hyal1, expression was under the control of the constitutive P_TK_ promoter [26,27]. Inducible protein expression could be a way to increase protein yield. For this reason, Hyal1 was expressed under the control of an inducible promotor. While isopropyl-β-d-thiogalactopyranoside (IPTG)-inducible recombinant protein expression based on the T7lac promotor results in high protein amounts, expression is prone to be leaky and turned out to be inappropriate for the surface display of Hyal1 (data not shown). Hence, rhamnose-controlled P_rha_ was selected as the promoter for this study. One advantage in comparison to, e.g., the P_BAD_ promoter, is the linear dependence of protein expression from the rhamnose concentration [31]. A plasmid for recombinant Hyal1 expression (pAIDAI_rha_-Hyal1) was constructed with features indicated in Figure 1 and a codon optimized DNA sequence of human Hyal1 [32,33]. In addition, a myc epitope was inserted at the C terminus of Hyal1 to enable the detection of protein expression by an anti-myc antibody (Figure 1).

**Figure 2:**
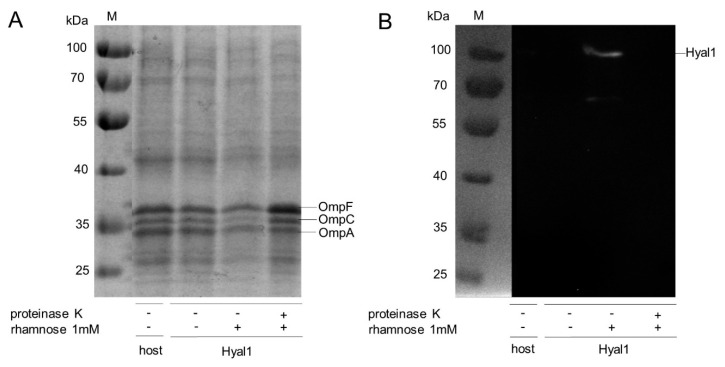
Expression of Hyal1 on *E. coli* F470 and protease accessibility test for proving surface display. Analysis of outer membrane protein isolations from *E. coli* F470 (host) and *E. coli* F470 carrying pAIDAI_rha_-Hyal1 without (−) and with (+) induction of Hyal1 fusion protein expression by addition of 1 mM rhamnose and proteinase K treatment prior to outer membrane protein isolation. Characteristic outer membrane proteins of *E. coli* F470 are indicated as OmpF, OmpC and OmpA. Apparent molecular weights of marker proteins are indicated on the left in kDa (M). 10% SDS-PAGE was stained with ProBlue Safe Stain^®^ (**A**), a primary monoclonal anti-myc antibody and secondary HRP-conjugated polyclonal antiserum were used for Western Blot analysis (**B**).

*E. coli* host strain F470 without plasmid as well as *E. coli* F470 carrying the plasmid pAIDAI_rha_-Hyal1 (Hyal1) were cultured to an OD_578_ of 0.4–0.6 and the expression of Hyal1 was induced by adding 1 mM rhamnose. Subsequently, outer membrane proteins were isolated [31] and analyzed by 10% sodium dodecyl sulfate-polyacrylamide gel electrophoresis (SDS-PAGE) [34]. The molecular weight of the Hyal1 fusion protein without CtxB signal peptide was calculated to be 96 kDa. In the SDS-PAGE, a protein band at this size was not visible in the outer membrane protein fraction (Figure 2A). Consequently, a corresponding Western Blot analysis was performed, with a primary monoclonal mouse anti-Hyal1 antibody and a secondary polyclonal HRP (horse radish peroxidase)-labeled rabbit anti-mouse antiserum. A clear band at around 100 kDa appeared, indicating the expression of Hyal1 fusion protein (Figure 2B). To prove the surface display, cells were treated with proteinase K after Hyal1 expression. Proteinase K as a protein is too large to pass the outer membrane. Consequently, only proteins displayed at the cell surface were accessible for proteolysis. Outer membrane protein A (OmpA), with a molecular weight of about 35 kDa, is a natural outer membrane protein of *E. coli*, with a domain in the periplasm that is linked to the murein layer. This periplasmic domain is degraded by proteinase K, in case the outer membrane is leaky. The membrane-embedded part of OmpA, which has a molecular weight of 25 kDa, is protected from degradation. Consequently, the appearance of OmpA at a full size molecular weight of 35 kDa and no additional band at 25 kDa is a clear indication of an intact outer membrane (Figure 2A, Lane 4). Hence, the degradation of Hyal1 under identical conditions, as seen in Figure 2B, Lane 4 indicates its successful surface display.

**Figure 3:**
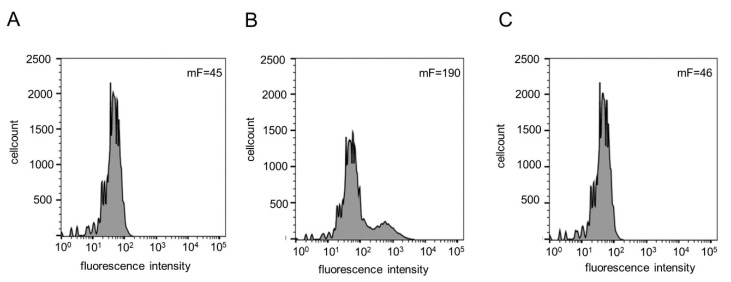
Flow cytometry analyses of immune-labeled *E. coli* F470 cells carrying pAIDAI_rha_-Hyal1. Analysis and determination of mean fluorescence intensity (mF) of cells without induction of Hyal1 expression (45) (**A**), induction of Hyal1 expression (190) (**B**) and cells with induced Hyal1 expression and proteinase K treatment prior to flow cytometry analysis (46) (**C**). After 16 h of cultivation cells were harvested and incubated with a primary monoclonal, anti-myc antibody and a secondary Dylight_633_-conjugated anti-IgG antiserum and analyzed after washing by flow cytometry.

For additional proof of the surface display, flow cytometry analysis was performed (Figure 3). Cells before and after induction of Hyal1 expression with rhamnose, as well as cells treated with proteinase K after induction were incubated with primary monoclonal mouse anti-myc antibody followed by an incubation with secondary Dylight_633_-conjugated rabbit anti-IgG mouse antiserum. A total of 50,000 cells per sample were analyzed *via* flow cytometry. The mean value of fluorescence (mF) of *E. coli* F470 pAIDAI_rha_-Hyal1 cells without induction of Hyal1 expression (control) was determined to be 45 (Figure 3A). The mF of cells after induction of Hyal1 expression was higher by a factor of four (mF = 190) (Figure 3B), whereas the mF of cells with Hyal1 expression, but treated with proteinase K, was 46—almost identical to the control cells (Figure 3C). These results clearly indicated the surface display of Hyal1 under the control of the inducible rhamnose promoter.

### 2.2. Influence of Induction Time on Hyal1 Expression and Activity

**Figure 4:**
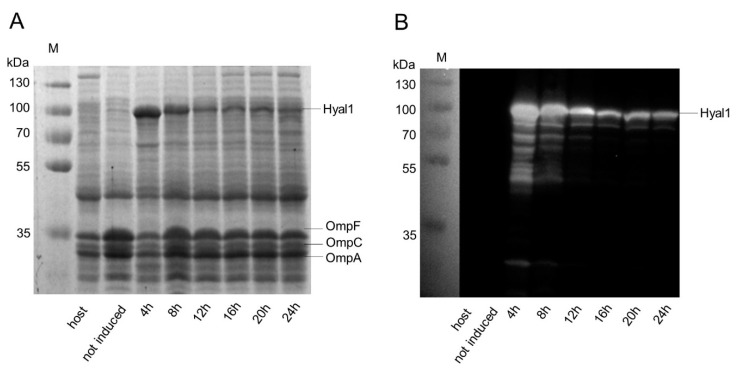
Induction time influences surface expression of Hyal1 on *E. coli* F470. Analysis of outer membrane protein isolations by 10% SDS-PAGE stained with ProBlue Safe Stain^®^ (**A**) and Western Blot (**B**) using a primary monoclonal, anti Hyal1 antibody and a secondary HRP-conjugated polyclonal antiserum. Apparent molecular weights of marker proteins are indicated on the left in kDa (M). Outer membrane proteins from *E. coli* F470 (host) and *E. coli* F470 carrying pAIDAI_rha_-Hyal1 with and without the induction of Hyal1 surface display by the addition of 1 mM rhamnose and increasing induction times. Protein samples were standardized by the amount of natural outer membrane proteins occurring, i.e., to show identical band sizes for OmpC, OmpF and OmpA.

The *E. coli* F470 host strain and seven cultures of *E. coli* F470 carrying pAIDAI_rha_-Hyal1 for inducible Hyal1 expression were cultured and, to six cultures, 1 mM rhamnose was added for induction of Hyal1 expression. The cultures were incubated for 4–24 h and kept at 4 °C after harvesting. Finally, outer membrane proteins were isolated and analyzed by SDS-PAGE and Western Blot as described above. *E. coli* F470 host and *E. coli* F470 cells carrying pAIDAI_rha_-Hyal1 without protein induction (controls) did not show any band corresponding to Hyal1 (Figure 4). Hyal1 fusion protein appeared as band around 100 kDa, visible in all samples after the addition of rhamnose (4–24 h). For this experiment, the total amount of protein loaded per sample was increased in comparison to the previous SDS-PAGE (Figure 2). This is indicated by the higher band intensity of the natural outer membrane proteins OmpF, OmpC and OmpA. [35]. The higher number of outer membrane proteins loaded also allowed Hyal1 fusion protein detection in SDS-PAGE. To our surprise, the yield of surface displayed Hyal1 decreased with an increasing induction time, although the total protein amount per lane of the SDS-PAGE was identical for each sample. A possible explanation could be the presence of OmpT, an outer membrane protease naturally occurring in *E. coli,* belonging to the aspartyl-protease family [36]. The release of surface displayed proteins by OmpT has been shown in previous studies [37,38,39]. OmpT could also be responsible for the additional bands occurring at lower molecular weights, most prominent after 4 and 8 h of induction. These additional bands could have been generated during outer membrane protein isolation as observed before [40]. ExPAsy peptide cutter online software was used to determine the possible cleavage sites of OmpT within the Hyal1 fusion protein [41]. In total, 29 cleavage sites at the N terminus of the Hyal1 fusion protein were predicted. Proteolysis of the membrane embedded ß-barrel at the C terminus could be excluded. A restriction of accessible cleavage sites due to stable secondary structures was not considered. A further R↓V cleavage site within the linker region between AA513 and AA514 of the Hyal1 fusion protein was described previously by Maurer et al. for a different passenger protein [37]. Consequently, we considered the decreasing amount of full length Hyal1, along with longer expression times, could be due to the release of Hyal1 into the supernatant due to OmpT cleavage. The degradation bands appearing at earlier time points, e.g., after 4 and 8 h, could be due to OmpT cleavage during outer membrane preparation or due to fragments of Hyal1 released by OmpT remaining in contact with the cell surface by uncleaved Hyal1, a phenomenon, that has been observed before in the case of surface displayed adrenodoxin [37] (Figure 4B).

**Figure 5:**
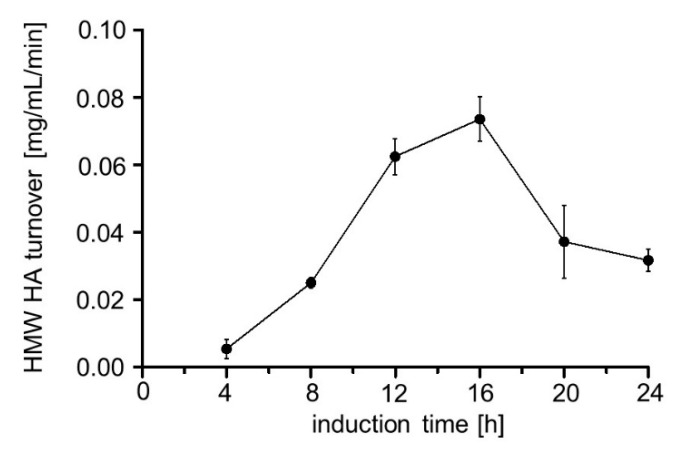
Enzyme activity of Hyal1 on the surface of *E. coli* F470, dependent upon the induction time. Enzyme activity determination was performed using the Stains-all assay [39]. Cells were cultured and incubated with 1 mM rhamnose for the induction times as indicated. Subsequently OD_578_ was set to 10 after washing with sodium formate buffer (100 mM) pH 3.5. hyaluronic acid (HA) was added (0.11 mg/mL final concentration) and reaction was performed for 1 min at 37 °C. Supernatants were analyzed for residual high molecular weight (HMW) HA using Stains-all. Mean values ± SD (n = 3).

Hyal1 enzyme activity was determined using a previously described Stains-all assay [42]. Interaction of Stains-all with HMW HA results in an absorbance at 650 nm, dependent on the concentration of HMW HA (Figure S1). Enzyme activity is commonly given in units (U), which corresponds to the µmols of the substrate converted per minute. Because the substrate molecules accepted by Hyal1 can be different, e.g., an octamer can be cleaved to a tetramer, followed by cleavage to a dimer, this is not appropriate for Hyal1 and does not allow us to determine enzyme kinetic measurements as required e.g., for a Lineweaver–Burk plot. Hence, the activity of Hyal1 is defined as the µmol-reducing sugar ends produced per minute. By the application of the Stains-all assay, only the decrease in HMW HA due to degradation by Hyal1 can be determined. For this purpose, the enzyme activity needs to be defined as the HMW HA turnover in mg/mL. The amount of degraded HMW HA by surface displayed Hyal1 was estimated with the help of a calibration curve (Figure S1). For the activity assay, cells were harvested and washed with sodium formate pH 3.5. Cell suspension was adjusted to an OD_578_ of 10 and HA was added to a final concentration of 0.11 mg/mL. After 1 min reaction time, Stains-all was added. Surprisingly, cells with the highest Hyal1 protein expression after 4 h of induction (Figure 4) showed the lowest HMW HA turnover, i.e., enzyme activity (Figure 5). The HMW HA turnover increased over time with decreasing protein amounts until it reached an optimum at 16 h of induction. Consequently, all further experiments were carried out after 16 h of induction time.

### 2.3. Influence of Rhamnose Concentration on Hyal1 Expression and Activity

**Figure 6:**
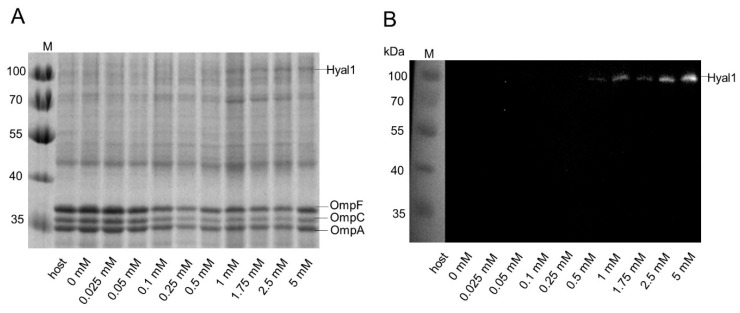
Surface expression of Hyal1 on *E. coli* F470, dependent upon inducer concentration. Outer membrane proteins of *E. coli* F470 (host) and *E. coli* F470 carrying pAIDAI_rha_-Hyal1 with and without (0 mM rhamnose) induction of Hyal1 protein expression were analyzed by 10% SDS-PAGE stained with ProBlue Safe Stain^®^ (**A**) and Western Blot analysis using a primary monoclonal anti-Hyal1 antibody and a secondary HRP-conjugated polyclonal antiserum (**B**). Apparent molecular weights are indicated on the left in kDa (M). Proteins samples were standardized by the amount of natural outer membrane proteins occurring, i.e., to show identical band size for OmpC, OmpF and OmpA. Rhamnose concentrations for induction of protein expression are given below the lanes of the gel and the western blot. Hyal1 and natural outer membrane proteins OmpF, C, and A are indicated.

*E. coli* F470 host cells (control) and 10 cultures of *E. coli* F470 carrying pAIDAI_rha_-Hyal1 were cultured and Hyal1 expression was induced by adding variant concentrations of rhamnose (0–5 mM). Protein expression was analyzed by SDS-PAGE and Western Blot analysis of the outer membrane protein preparations (Figure 6). The band corresponding to Hyal1 fusion at 100 kDa was not visible at rhamnose concentrations below 0.5 mM, but consecutively increased at higher concentrations of rhamnose. It needs to be emphasized that, in this experiment, the overall protein amount loaded per sample was lower than in the preceding experiment given in Figure 4. This led to fainter protein bands representing Hyal1.

**Figure 7:**
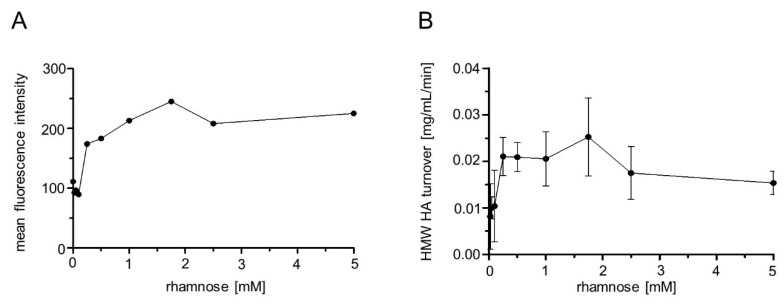
Mean fluorescence (mF) values of immunolabeled *E. coli* F470 carrying pAIDAI_rha_-Hyal1 (**A**) and HMW HA turnover (**B**), dependent upon the rhamnose concentration used for protein expression. For flow cytometer analysis, cells were harvested after 16 h induction time and incubated with a primary monoclonal anti-myc antibody and a secondary Dylight_633_ conjugated anti-IgG antiserum. 50,000 cells were analyzed per sample. Mean fluorescence intensity is plotted against rhamnose concentration. For HMW HA turnover, cells were harvested and washed with sodium formate buffer (100 mM) pH 3.5. Cell suspension was adjusted to an OD_578_ of 10. HA was added (0.11 mg/mL final concentration) and the reaction was run for 1 min at 37 °C. Supernatants were analyzed for residual HMW HA by Stains-all assay. Mean values ± SD (n = 3).

In addition, flow cytometry analysis of 50,000 cells per sample was performed as described above. Increasing rhamnose concentrations led to higher mF values, until a plateau was reached at 2 mM of rhamnose (Figure 7), which means that higher concentrations of inducer did not yield higher fluorescence. This observation is confirmed by earlier studies on protein expression under the control of a rhamnose promoter [31,43].

In addition, the HMW HA turnover, dependent upon the rhamnose concentration, was determined. Increasing concentrations of rhamnose were expected to yield higher Hyal1 expression and, hence, higher activity and HMW HA turnover. (Figure 7B). Cells with protein expression induced with rhamnose concentrations below 0.025 mM showed significantly less HMW HA turnover. However, a significant difference in the HA turnover at concentrations beyond higher 0.025 mM was not detectable. Finally, 1 mM rhamnose was maintained for inducing protein expression in all following experiments to be on the safe side. As already seen in the experiments described above, there was no correlation between the Hyal1 protein amount visible in the SDS-PAGE and enzymatic activity determined by Stains-all.

### 2.4. Influence of pH, Temperature, Substrate Concentration and Sodium Chloride on Enzyme Activity

**Figure 8:**
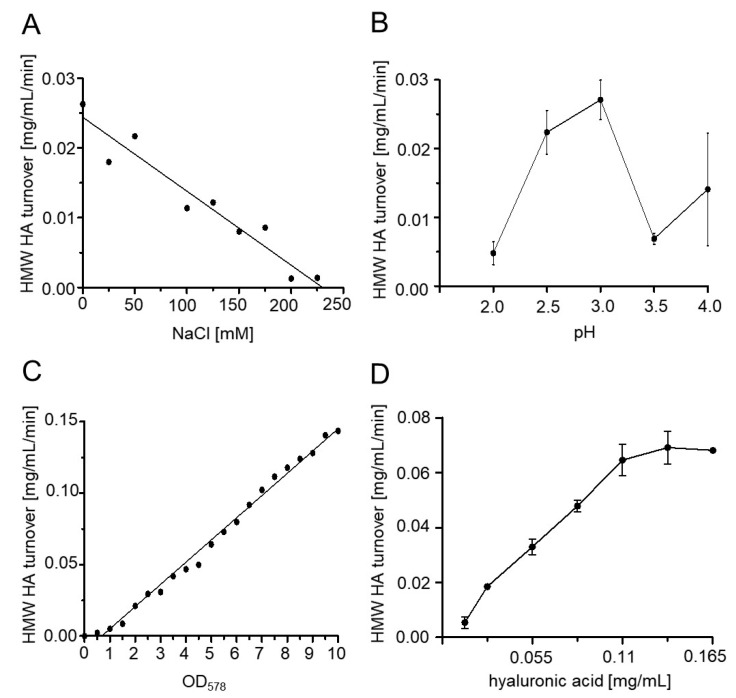
Improving the assay conditions for maximum Hyal1 activity. Whole cell activity measurements of cells displaying Hyal1 were performed. Cells were harvested after 16 h of induction and OD_578_ was set to 10 or as indicated, followed by incubation at 37 °C for one minute under different conditions. Enzyme activity as HMW HA turnover in mg/mL/min dependent on NaCl concentration (**A**) and pH value (**B**), optical cell density at 578 nm (**C**) and HA substrate concentration (**D**). Residual HMW HA was detected using the Stains-all assay. Mean values ± SD are given (n = 3).

*E. coli* F470 cells displaying Hyal1 were washed with sodium formate buffer and suspended in varying NaCl concentrations at varying pH and the desired OD_578_ was adjusted. Finally, hyaluronic acid diluted in appropriate sodium formate buffer was added (1:1 v/v). After one minute of reaction time, HMW HA turnover was measured using Stains-all. Hyal1 activity was strongly dependent on the NaCl concentration. The higher the NaCl concentration, the lower the turnover of high molecular weight HA (Figure 8A). Accordingly, all further experiments were performed using sodium formate buffer without any additional NaCl. HMW HA turnover was highest at pH 3.5 with a turnover of about 0.027 mg/mL HMW HA per min (Figure 8B). Hence, the pH optimum for Hyal1 was 3.5, which is in accordance with values in the literature [8]. A linear correlation was observed for HMW HA turnover and the number of bacterial cells displaying Hyal1. Increasing cell numbers led to an increased enzyme concentration and to an elevated HMW HA turnover (Figure 8C). An OD_578_ of five was finally chosen as appropriate for inhibitor screening. Low amounts of bacterial cells may reduce probable site effects, like interactions between a potential inhibitor and the surface of a single bacterial cell. The HMW HA turnover was determined to be dependent upon varying HA concentrations. Turnover increased linearly up to 0.11 mg/mL HA and reached a plateau at higher concentrations (Figure 8D). Hence, a HA concentration of 0.11 mg/mL was chosen as substrate concentration for all following experiments.

### 2.5. Reaction Time

**Figure 9:**
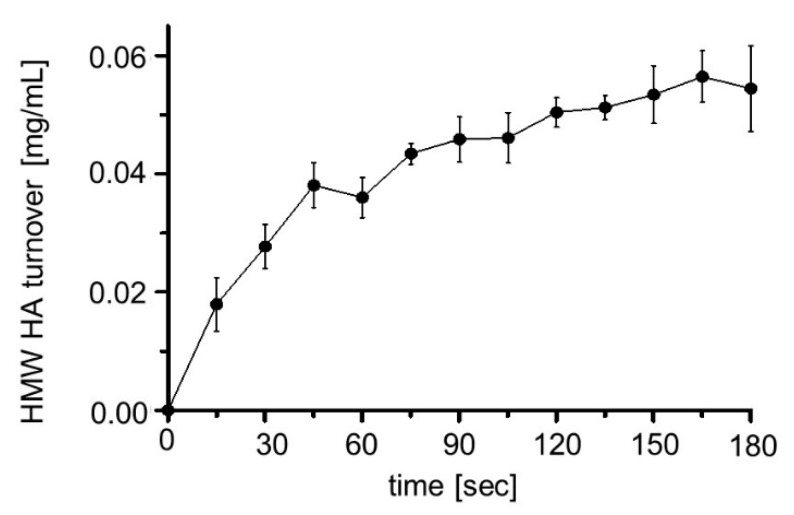
HMW turnover depending on the reaction time. *E. coli* F470 cells expressing Hyal1 were cultivated and after 16 h of induction the OD_578_ was set to 10 by adding reaction buffer (sodium formate buffer (100 mM) pH 3.5, without NaCl). After adding 0.22 mg/mL HMW HA (1:1 v/v) reaction was performed at 37 °C for various times. The residual HMW HA was detected using the Stains-all assay. Mean values ± SD are given (n = 3).

Because inhibitor testing should be done under linear reaction conditions, the enzyme kinetics of HA turnover were determined. For this purpose, the initial HMW HA turnover of a cell suspension with an OD_578_ = 10 was determined after induction of protein expression with 1 mM rhamnose for 16 h. The reaction was followed for 180 s by determining the residual amount of HMW HA in the cell supernatant by the Stains-all assay (Figure 9). It turned out that the HMW HA turnover rate increased in a linear way until 45 s of reaction. At later time points, the curve appeared to bend. Consequently, for inhibitor screening, the reaction time was set at 30 s, in order to be in the linear range.

### 2.6. Inhibitor Screening

**Figure 10:**
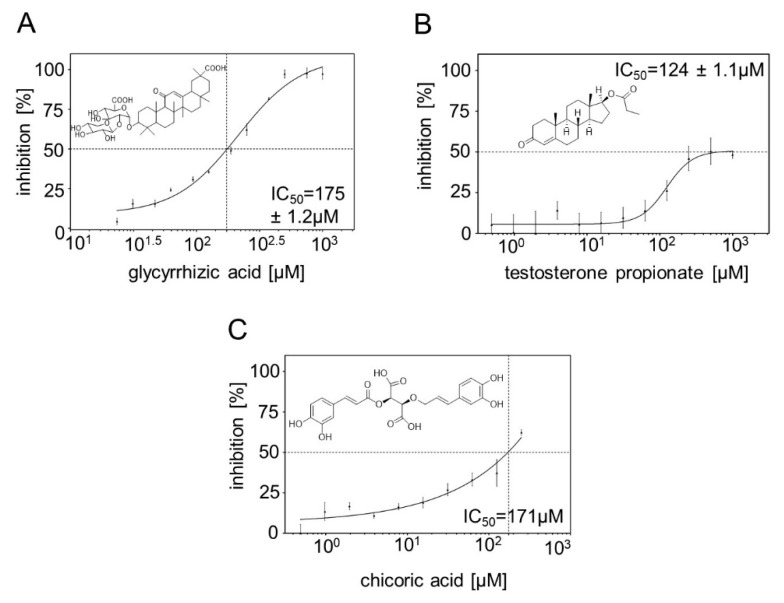
IC_50_ curves of glycyrrhizic acid (**A**), testosterone propionate (**B**) and chicoric acid (**C**). Inhibition of Hyal1 was tested using 12 different concentrations of testosterone propionate and glycyrrhizic acid and 10 different chicoric acid concentrations. The horizontal dotted line marks represent 50% inhibition. The vertical dotted line marks represent the inhibitor concentration at 50% inhibition. IC_50_ value of 175 ± 1.2 µM for glycyrrhizic acid, 124 ± 1.1 µM for testosterone propionate and 171 µM for chicoric acid were determined. Mean values ± SD are given (n = 3). Because of the incomplete course of the curve obtained for chicoric acid, no SD could be calculated.

Two well-known Hyal1 inhibitors described previously are Vcpal and glycyrrhizic acid. These compounds were tested on the inhibition of surface displayed Hyal1. Inhibition by Vcpal at a concentration of 200 µM was rudimentary (18.5%). Consequently, no IC_50_ value was determined. Glycyrrhizic acid showed more than 90% inhibition at a concentration of 200 µM. Hence, the IC_50_ value was determined and turned out to be 175 ± 1.2 µM (Figure 10). This is in agreement with previous studies [24].

Steroids are known to have an effect on HA metabolism [44,45,46]. Therefore, 19 different testosterone derivatives were tested on Hyal1 inhibition (Tables S1 and S2) [44,45,46]. In addition, phenylpropanoids, in particular, cinnamic acid derivatives, have been shown before to have inhibitory effects on BTH [44]. For this reason, 15 cinnamic acid derivatives were tested on Hyal1 inhibitions as well (Table S3) [47]. Out of these compounds, testosterone propionate and chicoric acid showed the best inhibition values with 47% and 40% at 200 µM and 100 µM, respectively. Unfortunately, the Stains-all assay was strongly impaired by high concentrations of chicoric acid beyond 250 µM. Consequently, 250 µM was the highest concentration that could be used in the IC_50_ value determination, but it resulted in a Hyal1 inhibition below 100%. This is a considerable restraint, and the IC_50_ value, determined to be 171 µM, has to be considered with reservations. Due to the incomplete course of the curve, the standard deviation for this IC_50_ value could not be determined. With testosterone propionate, it was not possible to obtain more than 50% inhibition, regardless of the concentrations applied. This could be an indication of a mixed type or partial inhibition of Hyal1 by this compound. Because the curve showed a typical, sigmoidal course, a relative IC_50_ value could be determined, referring to the concentration required for half maximum inhibition, which turned out to be 124 ± 1.1 µM (Figure 10).

## 3. Discussion

Production of recombinant human Hyal1 has been a bottleneck for inhibitor screening for a long time. Several expression systems have been studied in the past. *E. coli* and *Drosophila* Schneider 2 (DS-2) cells as potential systems were investigated and compared by Hofinger et al. [27]. Cytosolic expression in *E. coli* resulted in high protein yields but very low enzyme activity. Enzyme expression in DS-2 cells was time consuming and the final amount of Hyal1 was less than that obtained with *E. coli*. However, specific enzyme activity was 75 times higher in eukaryotic cells [28]. For mechanistic studies, enzyme kinetics or inhibitor screening the bovine enzyme BTH was used instead [22,23,48,49]. BTH is commercially available, and has a sequence identity of only 40% with human Hyal1 (BTH, UniProtKB Q7YS45, Hyal1, UniProtKB Q12794) [50,51]. The constitutive expression of Hyal1 on the surface of *E. coli* F470 as a more reliable enzyme source was published before [29]. Activity of hyaluronidases are usually given in units (U), defined as 1 µmol reducing sugar end per minute. In previous studies, we determined the activity of surface displayed Hyal1 to be 4.54 × 10^−6^ mU per single cell, by comparing the HMW HA turnover with that of known U from commercially available ovine testicular hyaluronidase (OTH). In this study, the expression of Hyal1 on the surface of *E. coli* F470 under control of an inducible P_rha_ promoter led to an increase in activity by a factor of 100, resulting in 6.8 × 10^−4^ mU per single cell, in comparison with cells displaying Hyal1 with constitutive expression (4.6 × 10^−6^ mU) [28]. We also purified Hyal1 after expression in BTI-Tn-5B1-4 insect cells (High Five^TM^ cells). The enzyme showed a specific activity of 20.8 mU/mg and similar pH optimum and NaCl dependency as the surface displayed enzyme (Figure 8). However, the amount of enzyme obtained by this approach was not sufficient for inhibitor testing. As shown for surface displayed Hyal1 here, enzyme expression was strongly dependent on the induction time. The amount of Hyal1 decreased with longer induction times due to the release of Hyal1 fusion protein by OmpT, an outer membrane protease (Figure 4). This clearly points to a flaw in the current approach. Because Hyal1 expression level appears to be sensitive to the induction time and other factors, this needs to be monitored carefully, because it may affect the accuracy of the IC_50_ value determination.

The rhamnose-dependent expression of a gene of interest using P_rha_ promotor has been described before and worked well for Hyal1 expression (Figure 6) [31,43]. In accordance, Hyal1 activity increased with increasing inducer concentrations, attaining a plateau at 0.02 mg/mL/min HMW HA turnover (Figure 7B).

Parameters such as pH and salt concentration can have a strong influence on enzymatic activity. As Hyal1 is located in the lysosomes in the human body, highest enzyme activity is expected at acidic pH levels. In addition, optimal pH was determined to be 3.5 in many studies before [26,28,52]. In this study, Hyal1 was most active at pH 3.5 which was in good accordance with the values found in the literature. NaCl concentration was evaluated to have an influence on Hyal1 activity in former studies as well. Hyal1 expressed and purified from *E. coli* showed the highest activity without the addition of NaCl to the reaction buffer [28]. This was confirmed by the results of our study, which showed decreasing Hyal1 activity with an increase in NaCl concentration.

**Figure 11:**
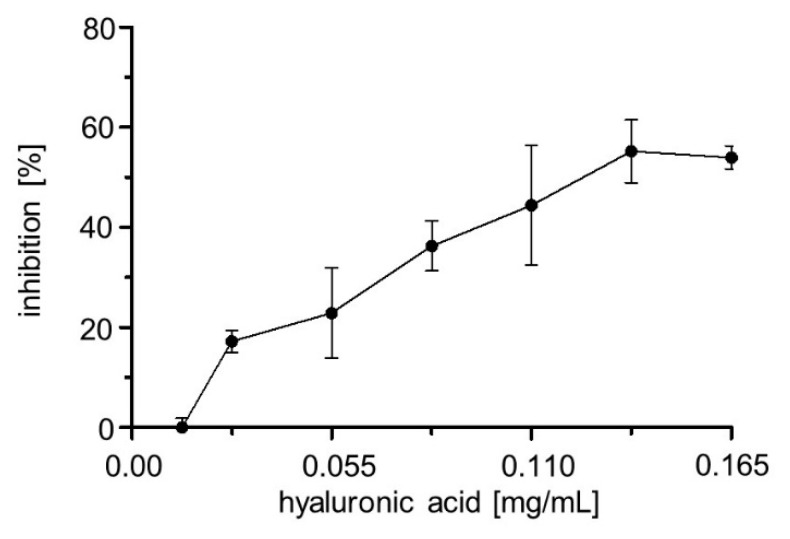
Inhibition of Hyal1 by 250 µM glycyrrhizic acid, in dependency on HMW HA concentration. *E. coli* F470 cells expressing Hyal1 were harvested after 16 h of induction and the OD_578_ was set to 10 adding reaction buffer (sodium formate pH 3.5, 0 mM NaCl). Glycyrrhizic acid was added [250 µM final) and cells were preincubated for 10 min. After adding 0.22mg/mL HMW HA, a (1:1 v/v) reaction was performed at 37 °C for one minute. Inhibition of Hyal1 was calculated in accordance to our previous studies. Mean values ± SD (n = 3).

A lot of different compounds from diverse substance classes and various origins were tested for inhibiting hyaluronidases from different sources. Heparin, as a representative for the class of sulfated glycosaminoglucans, was found to inhibit BTH almost 30 years ago (IC_50_ = 17 µM) [53]. Isoyama et al. published 21 additional hyaluronidase inhibitors belonging to the class of polystyrene 4 sulfonates (PSS) and O-sulfated HA derivatives [24]. IC_50_ values for Hyal1 were determined to be in the nanomolar and submicromolar range (PSS 990,000, IC_50_ = 0.0096 µM; sHA2.0, IC_50_ = 0.0096 µM; heparin, IC_50_ = 0.39 µM). These polymers showed inhibition towards different hyaluronidases. Although such polymers could be a good starting point for inhibitor screening, testing in the Stains-all assay is not possible due to polymer–dye interactions, that led to errors in the photometric readout, and, hence, heparin or other polymers could not be tested as Hyal1 inhibitors in this study. The classes of indole carboxamides and salicylates were examined as Hyal1 inhibitors before [54,55], with considerable inhibition of BTH. A large portion of hyaluronidase inhibitors identified are natural compounds belonging to diverse substance classes, such as the triterpene-containing saponines, flavonoids, isoflavonoids or even rubromycins [22,56,57,58]. Glycyrrhizic acid is the best investigated Hyal1 inhibitor to date. Therefore, it was used as reference inhibitor here. The IC_50_ value of glycyrrhizic acid was determined to be 175 ± 1,2 µM, which was in the same order of magnitude as described before, and almost identical as the IC_50_ value determined with surface displayed Hyal1 using a constitutive expression system (177 µM) [24,28,29]. To get a closer view of the binding of glycyrrhizic acid to Hyal1 (PDB: 2PE4), docking studies were performed. These were adjusted to low pH surroundings and by predicting the lowest energy levels, the best docking scores for glycyrrhizic acid binding was not obtained at the active site. This *in silico* experiment pointed to an allosteric inhibition of Hyal1 by glycyrrhizic acid. This could be supported experimentally by activity measurements using the Stains-all assay. For this purpose, inhibition by glycyrrhizic acid was measured at different substrate (HMW HA) concentrations and, subsequently, inhibition in % was plotted against substrate concentration (Figure 11). To our surprise, inhibition of Hyal1 by glycyrrhizic acid at a constant concentration of 250 µM increased with increasing amounts of HMW HA. This effect fits the *in silico* results and could point on an uncompetitive mode of inhibition. Unfortunately, as outlined above, the Stains-all assay cannot be used to determine a Hyal1 kinetics as required, e.g., for a Lineweaver–Burk plot. Consequently, these results cannot be seen as more than a first hint that glycyrrhizic acid is not a competitive inhibitor, and need confirmation through further experiments. The mode of inhibition for glycyrrhizic acid has not been determined before for human Hyal1, but it has for bacterial hyaluronidases, where it was reported be uncompetitive [56], however these enzymes belong to a different class than human Hyal1.

Two new Hyal1 inhibitors were identified in this study. Testosterone propionate, a steroid derivative, was determined to be moderately active against Hyal1 with an IC_50_ value of 124 ± 1.1 µM. Regulation of HA metabolism by steroids has been previously described [44,46,59]. Tanyildizi et al. investigated the influence of progesterone and testosterone on hyaluronidase activity in sheep, but came to the conclusion that the compounds were not inhibitorily active. Tranilast, a modified cinnamic acid was identified as a weak hyaluronidase inhibitor in a previous study [60]. Several cinnamides and cinnamic acid derivatives, belonging to the class of phenylpropanoids, were tested here (Table S3). Out of these compounds, only chicoric acid turned out to be active against Hyal1 with an IC_50_ value of 171 µM (Figure 10).

## 4. Conclusions

An improved whole cell assay with surface displayed Hyal1 for the screening assay was established and led to the identification of new inhibitors. The IC_50_ values of chicoric acid and testosterone propionate were in the same order of magnitude as that of the known reference inhibitor, glycyrrhizic acid.

## 5. Materials and Methods

### 5.1. Chemicals

Chemicals for cultivation of *E. coli* F470 were purchased from Roth, Karlsruhe, Germany. Including yeast extract, tryptone, agar, etc. Vera Seq Polymerase was purchased from Biozym (Hessisch Oldendorf, Germany). Reagents for Stains-all assay and reaction buffer were purchased from Merck, Darmstadt, Germany. *E. coli* F470 was kindly provided by J. Seydel (Center for Medicine and Biosciences, Biophysics, Borstel Research Center, Sülfeld, Germany) and used for expression experiments.

### 5.2. Plasmid Construction

Artificial synthesized genes for the *E. coli* codon optimized reading frame of Hyal1 were purchased from GeneArt (Regensburg, Germany). The ligase free In Fusion cloning method was applied for passenger exchange of pPQ62 to obtain the plasmid pAIDAI_rha_ Hyal1 [32,33]. The following primers 5′TCATCTCAGAAGAGGATCTGGGTACCCTTAATCCTACAAAAG AAAGTGC3′ (forward), 5′CAGCGGACCACGAAAGGTGATGTTCTGCGGAGTACCGTGAGCGT3′ (reverse) for amplifi-cation of the backbone of pPQ62 and Hyal1 5′TTTCGTGGTCCGCTG3′ (forward), 5′CAGATCCTCTTCTGAGATGAGTTTTTGTTCCCACATGCTTTTACGTTC3′ (reverse), with an additional sequence insertion of an anti-myc epitope tag at its 3′ end, were amplified by PCR. *E. coli* stellar were used for cloning experiments. The coding region for Hyal1 fusion protein was controlled by DNA sequence analysis.

### 5.3. Escherichia coli F470 Culturing

*E. coli* F470 cells with and without the plasmid pAIDAI_rha_ Hyal1 were cultivated for 16 h at 37 °C and 200 rpm in LB (lysogeny broth) medium as a pre-culture containing carbenicillin (1:100) if necessary. Subsequently, fresh LB medium was inoculated with pre-culture (1:100). The cells were grown up to an OD_578_ of 0.4 to 0.6 and induced with desired rhamnose concentrations (0–5 mM). Cell suspension was cultivated for 4–24 h, at 37 °C and 200 rpm. Cells were harvested by centrifugation at 4 °C, 3500× *g* for 5 min and washed with reaction buffer (sodium formate buffer, 100 mM, pH 3.5) or phosphate buffered saline (PBS) as needed for further experiments.

### 5.4. Preparation of Outer Membrane Proteins

The outer membrane protein isolation for 80 mL of the main culture was performed by a modified method according to Park et al. [34]. Cells were harvested and washed with 3 mL 0.2 M Tris/HCl pH 8.0. Prior to outer membrane protein isolation, one culture expressing Hyal1 was treated with proteinase K with 50 m Anson units to determine cell integrity. Cell suspension was incubated for 1 h at 37 °C. The reaction was stopped by adding phenylmethylsulfonyl fluoride (PMSF) (1 mM) and cells were washed with 0.2 M Tris/HCl pH 8.0. After harvest, all cells were suspended in 3 mL 0.2 M Tris/HCl pH 8.0 and 6.4 mL H_2_O and lysed by adding lysozyme (200 µg/mL final concentration), sucrose (20 mM final concentration), 0.1 mM ethylene diamine triacetate (EDTA) (final) and were incubated for 10 min at room temperature (RT). Subsequently, PMSF (0.5 mM final) and aprotinin (10 µg/mL) were added. By the addition of 10 mL extraction buffer (50 mM Tris/HCl, 2% Triton X 100, 10 mM MgCl_2_) and DNAse (10 µg/mL final), the outer membranes were isolated after incubation for 30 min on ice. The suspensions were centrifuged at 4500× *g* for 10 min at 4 °C and the supernatants were centrifuged for 38,700× *g* for 10 min at 4 °C and washed with water twice. The outer membrane protein isolations were dissolved in water for SDS-PAGE and Western Blot analyses.

### 5.5. SDS-PAGE and Western Blot Analysis

The outer membrane protein isolations were diluted (1:2) with sample buffer containing 10 mM Tris/HCl (pH 6.8, 0.2% bromphenolblue, 4% SDS, 20% Glycerol, 200 mM dithiothreitol, DTT). The samples were boiled for 20 min at 95 °C and analyzed by a 10% SDS-PAGE. After staining with ProBlue Safe Stain^®^, the molecular weight of the proteins was confirmed using pre-stained molecular weight markers. For Western Blot analysis the proteins were transferred to a polyvinylidene fluoride (PVDF) membrane under a constant voltage of 100 V. The membrane was blocked by PBS 0.1% Tween 20 containing 5% milk powder at RT for 45 min. The primary antibody was incubated for 1 h (mouse anti-myc antibody or mouse anti-Hyal1 antibody 1:200) at RT. After washing, the secondary rabbit anti-mouse IgG HRP-labeled antiserum was added (1:10,000) and incubated for additional 2 h at RT. The membrane was incubated with HRP substrate and protein bands visualized using Chemocam HR16 camera system (Intas, Göttingen, Germany).

### 5.6. Flow Cytometry Analysis

*E. coli* F470 cells were harvested (4500× *g* for 5 min) after cultivation and washed with filter-sterilized PBS. 1 mL of cell suspension was set to an OD_578_ of 0.2 and centrifuged. The pellet was suspended in 500 µL PBS containing 3% BSA and incubated for 15 min at RT. Cells were centrifuged and suspended in 100 µL of an anti-myc antibody solution (1:1 in PBS). After incubation for 45 min at RT, the cells were washed three times and incubated with 100 µL goat anti-mouse Dylight_633_-labeled antiserum solution. After additional incubation for 1 h at RT, the cells were washed three times and analyzed by FACS Aria III (BD, Heidelberg, Germany).

### 5.7. Stains-All Assay

A slightly modified Stains-all assay was performed as described in our previous studies [28]. *E. coli* F470 cells were cultivated as previously described and washed using the reaction buffer. The OD_578_ was set to 10 and inhibitors were solved in dimethylsulfoxid (DMSO) (appropriate concentrations were added). After 10 min of preincubation, the 0.22 mg/mL HA was added to obtain a final HA concentration of 0.11 mg/mL and an OD_578_ of five. After the desired reaction time, the cell suspension was centrifuged, and the supernatant was analyzed at 650 nm after adding Stains-all reagent. The inhibition values were calculated as previous described [28].

### 5.8. In Silico Experiments

To elucidate the possible binding mode of the glycyrrhizic acid further, molecular docking experiments against Hyal1 were performed, utilizing the software “Molecular operating environment (MOE)” supplied by the Chemical Computing Group (CCG, Montreal, Canada) (MOE, 2017). Initially, a structural model of Hyal-1 was acquired from the Protein Data Bank (PDB-ID 2PE4) (RCSB). Subsequently, the protein structure was corrected in MOE (correcting protonation states as well as terminal, faulty or misaligned amino acids) and energy minimized using the MMFF94x force field [61]. Glycyrrhizic acid was subsequently analyzed concerning its possible interaction with Hyal1 by performing molecular docking experiments against all atoms of the prepared protein structure.

## Supplementary Materials

The following are available online at https://www.mdpi.com/1424-8247/13/4/54/s1, Figure S1: Calibration curve—Stains-all method. Table S1: Structure and inhibition values of testosterone derivatives, Table S2: Structure and inhibition values of dihydrotestosterone derivatives, Table S3: Structure and inhibition values of cinnamic acid derivates.
