# Supplementary material for: Improved Surface Display of Human Hyal1 and Identification of Testosterone Propionate and Chicoric Acid as New Inhibitors
Source: Pharmaceuticals (Basel). 2020 Mar 26;13(4):54. doi: 10.3390/ph13040054 (PMC7243119; doi:10.3390/ph13040054)
Supplement: Supplementary file 1 [file pharmaceuticals-13-00054-s001.pdf]

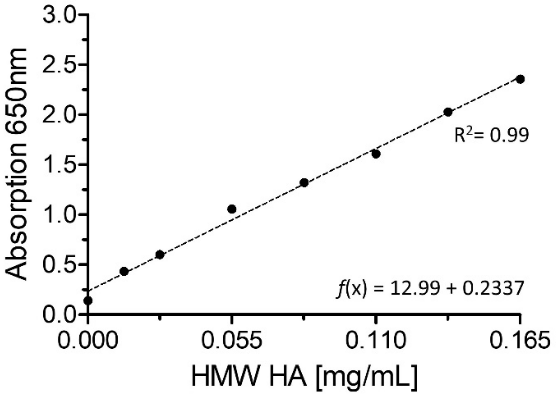

Figure S 1: Calibration curve – Stains-all method. Absorption depending on HMW HA [mg/mL].

Table S 1: Structure and inhibition values of testosterone derivatives

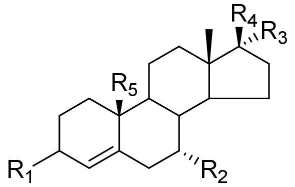

	R1	R2	R3	R 4	R5	inhibition [%] 200 µM
YC338	O	H	H	OH	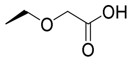	8
MR241	O	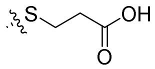	H	OH	CH_3	0
MR234	O	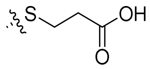	OH	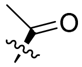	CH_3	0
YC208	O	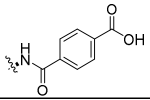	H	OH	CH_3	22
EM383	O	H	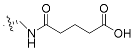	OH	CH_3	6
MR123	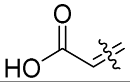	H	H	OH	CH_3	22
3CMO-Testo	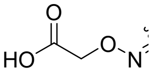	H	H	OH	CH_3	28
EM892	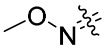	H	H	OH	CH_3	20
EM814	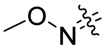	H	H	OH	H	17
Testo-3CMOesterMe	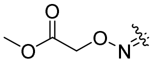	H	H	OH	CH_3	17
YC141	O	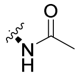	H	OH	CH_3	0
MR497	O	H	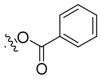	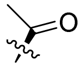	CH_3	0
MR405	O	H	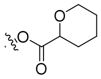	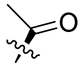	CH_3	13

Table S 2: Structure and inhibition values of dihydrotestosterone derivatives

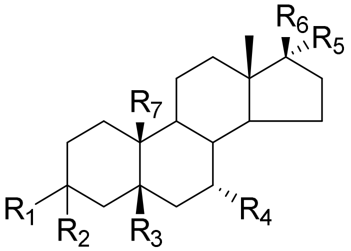

	R1	R2	R3	R4	R5	R6	R7	Inhibition [%] 200 μM
3CMO-DHT	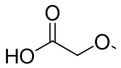	-	H	H	H	OH	CH_3	31
EM817	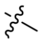	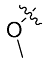	H	H	H	OH	CH_3	3
MR22	O	-	H	H	H	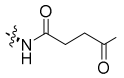	CH_3	0
MR568	O	-	H	H	H	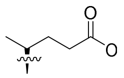	CH_3	0
MR572	O	-	H	H	H	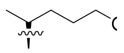	CH_3	5
MR574	O	-	H	H	H	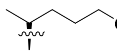	CH_3	18

Table S 3: Structure and inhibition values of cinnamic acid derivates

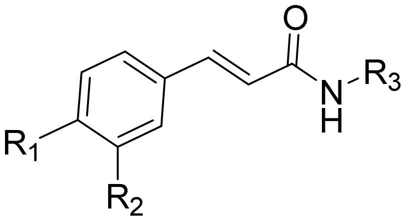

	R1	R2	R3	inhibition [%] 200 μM
SB12 + LR78	OH	H	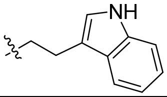	30
SB14	OH	H	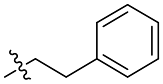	25
SB15	OH	H	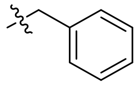	26
SB16 + SB22	OH	H	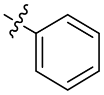	27
SB25	OH	H	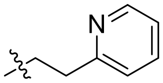	37
SB18	OH	H	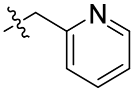	19
SB21	OH	H	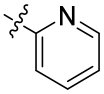	33
SB19 + SB20 + SB23	OH	H	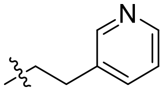	18
SB26	OH	H	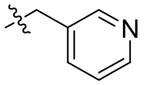	20
SB27	OH	H	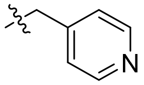	4
LR76	H	H	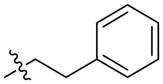	11
LR 77	H	H	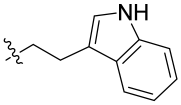	0
LR80	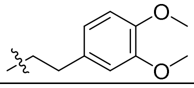	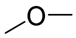	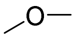	0
SB9	H	H	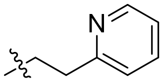	0
